# Absorption and Tissue Distribution of Siphonaxanthin from Green Algae

**DOI:** 10.3390/md18060291

**Published:** 2020-06-01

**Authors:** Zhuosi Li, Jiawen Zheng, Xiaolin Luo, Yuki Manabe, Takashi Hirata, Tatsuya Sugawara

**Affiliations:** 1 Division of Applied Biosciences, Graduate School of Agriculture, Kyoto University, Kyoto 6068502, Japan; lizhuosi624@gmail.com (Z.L.); feitianmao0715@gmail.com (J.Z.); shelyluo@gmail.com (X.L.); manabe.yuki.8c@kyoto-u.ac.jp (Y.M.); hiratan@mbox.kyoto-inet.or.jp (T.H.); 2 Department of Rehabilitation, Shijonawate Gakuen University, Osaka 5740011, Japan

**Keywords:** siphonaxanthin, dehydro-metabolite, white adipose tissue, metabolic pathway in vivo

## Abstract

Siphonaxanthin has been known to possess inhibitory effects against obesity, inflammation, and angiogenesis. However, little information on its in vivo bioavailability and biotransformation is available. To assess the bioavailability and metabolism of siphonaxanthin, its absorption and accumulation were evaluated using intestinal Caco-2 cells and Institute of Cancer Research (ICR) mice. Siphonaxanthin was absorbed and exhibited non-uniform accumulation and distribution patterns in tissues of ICR mice. Notably, in addition to siphonaxanthin, three main compounds were detected following dietary administration of siphonaxanthin. Because the compounds showed changes on mass spectra compared with that of siphonaxanthin, they were presumed to be metabolites of siphonaxanthin in ICR mice. Siphonaxanthin mainly accumulated in stomach and small intestine, while putative metabolites of siphonaxanthin mainly accumulated in liver and adipose tissues. Furthermore, siphonaxanthin and its putative metabolites selectively accumulated in white adipose tissue (WAT), especially mesenteric WAT. These results provide useful evidence regarding the in vivo bioactivity of siphonaxanthin. In particular, the results regarding the specific accumulation of siphonaxanthin and its metabolites in WAT have important implications for understanding their anti-obesity effects and regulatory roles in lipid metabolism.

## 1. Introduction

Carotenoids are structurally and functionally an diverse group of natural pigments of the polyene type. Carotenoids with a structure of conjugated double bonds are known to be extremely potent natural antioxidants because of their ability to physically and chemically quench singlet oxygen and scavenge other reactive oxygen species [1,2]. The antioxidant potential of carotenoids is of particular significance to human health, owing to their ameliorative effects on oxidative stress, an essential contributor to the pathogenic processes of many diseases [3,4]. For example, many carotenoids with great antioxidant properties displayed a risk reduction in chronic inflammatory diseases such as preventing inflammation and insulin resistance, providing protection against UV light damage and age-related diseases, and promoting the immune response in the liver, kidneys, and eyes [5].

Several factors influence the bioavailability, absorption, transport, metabolism, and accumulation of dietary carotenoids. Current research has been focused on exploring the potential of carotenoids in human health and elucidating important aspects regarding the digestion, absorption, and metabolism of carotenoids. The degree of food matrix disruption and other dietary components could affect the bioavailability of carotenoids [6]. For instance, the efficiency of carotenoid absorption in the digestive tract can be enhanced by food processing and additional oil and fat, while the coexistence of dietary fibers has been known to suppress their absorption [7]. Other factors, such as genetic factors, gender, age, and nutritional status, also affect the bioavailability of carotenoids. It has been suggested that the absorption of most carotenoids involves several steps, including the release of carotenoids from the food matrix by digestion, dispersion into lipid emulsion particles, incorporation into mixed micelles, uptake by intestinal cells, and secretion into the lymphatic system as lipoprotein particles [3,7,8]. Next, carotenoids are exclusively transported in plasma by lipoproteins and are further distributed in tissues [9].

Polar xanthophylls, such as astaxanthin, fucoxanthin, and siphonaxanthin, which are generally present in aquatic organisms, have been shown to possess beneficial bioactivity in animal models or humans [5]. Siphonaxanthin is a marine carotenoid and an oxidative metabolite of lutein, possessing a structure similar to lutein, except for one keto group located at C-8 and an extra hydroxyl group at C-19. Siphonaxanthin is a special xanthophyll found in green algae, such as *Caulerpa lentillifera*, *Codium fragile,* and *Codium cylindricum* [10].

**Figure 1:**
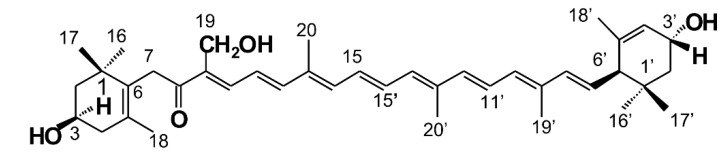
Chemical structure of siphonaxanthin (3,19,3’-Trihydroxy-7,8-dihydro-β,ε-caroten-8-one).

Siphonaxanthin (Figure 1) has been shown to possesses several potential bioactivities [11]. In vitro siphonaxanthin potently inhibits angiogenesis in vascular endothelial cells and induces apoptosis in human leukemia (HL-60) cells [12,13]. Additionally, siphonaxanthin can modulate inflammatory responses and suppress advanced glycation end product-induced inflammatory responses in vitro [14,15]. In particular, we have focused on the regulatory effect of siphonaxanthin on lipid metabolism. In vitro, we have observed that siphonaxanthin powerfully inhibits lipogenesis both in 3T3-L1 cells [16] and hepatocytes [17]. In vivo, siphonaxanthin has shown inhibitory effects on lipid accumulation in obese mice [16,18]. Furthermore, siphonaxanthin protects Ob/Ob mice fed a high-fat diet against lipotoxicity by ameliorating somatic stress and restoring the anti-oxidative capacity [19]. Thus, we hypothesized that the bioavailability and biotransformation of siphonaxanthin in vivo would allow its use for a dietary supplementation.

In this study, we first evaluated the absorption and biotransformation of siphonaxanthin using intestinal Caco-2 cells, which are widely used as a model to study and predict intestinal absorption and the transport of compounds at an early stage of drug or supplement development [20]. To understand the in vivo metabolism of siphonaxanthin, we investigated the tissue distribution, metabolic transformation, and accumulation of dietary siphonaxanthin using ICR mice [21]. This study will be useful for developing the applications of siphonaxanthin as a functional food. In particular, this study provides important reference information to further improve our knowledge regarding the anti-obesity effect of siphonaxanthin.

## 2. Results

### 2.1. Uptake of Siphonaxanthin by Caco-2 Cells

**Figure 2:**
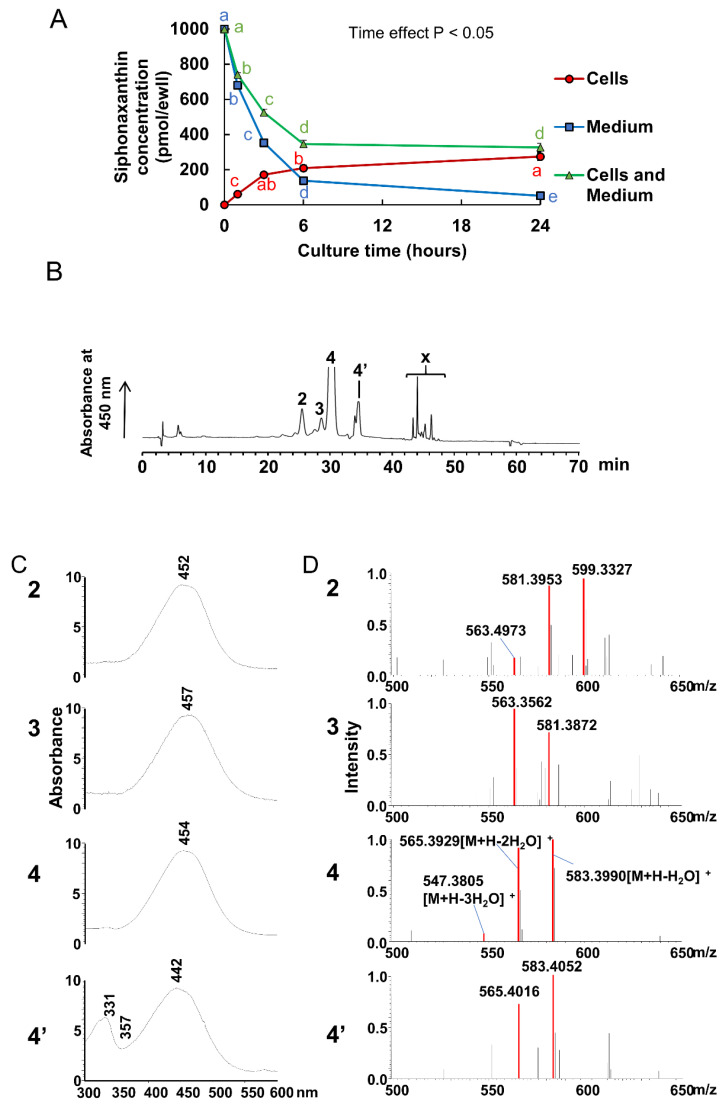
Uptake and metabolite analysis of siphonaxanthin in Caco-2 cells (**A**) Changes in siphonaxanthin concentration in Caco-2 cells and medium. Values are means ± SEM, n = 3. Data were analyzed by 1-factor ANOVA with repeated measures. “Time effect *p* < 0.05” indicates that there are differences in siphonaxanthin concentration variables at each time point within every group (cells, medium and cell+medium groups). Mean values without the same letter label indicate that they are significantly different within cells (red), medium (blue), or medium and cells (green), respectively, *p* < 0.05. (**B**) Representative HPLC chromatograms of the extract from Caco-2 cells after treatment with siphonaxanthin-containing micelles for 24 h. HPLC analysis was performed as described in the experimental methods. Peaks: 2, 3, and x, unknown metabolites; 4, siphonaxanthin; 4′, cis isomer of siphonaxanthin. Retention time: peak 2 at 25 min, peak 3 at 28 min, peak 4 at 30 min and peak x at 43–48 min. In the Caco-2 cells, we did not detect the peak corresponding to the peak 1 in the Figure 4 (mice data). Thus, in order to compare the corresponding peak at the same retention time compared with the Figure 4, here, the label “1” were not used. The detection wavelength was 450 nm. UV-vis spectra (**C**) and APCI-MS spectra (**D**) of peaks 2, 3, 4, and 4’. LC-MS with APCI analysis was performed as described in the experimental methods. HPLC, High-performance liquid chromatography; APCI, Atmospheric pressure chemical ionization; LC, Liquid chromatography; MS, Mass spectrometry.

First, the uptake of siphonaxanthin was evaluated in Caco-2 cells as a model for intestinal epithelial absorption. Caco-2 cells were incubated with 1 nmol/well of siphonaxanthin solubilized micelles for 1, 3, 6, and 24 h. The concentration of siphonaxanthin in the cells and medium was quantified (Figure 2A). The cellular concentration of siphonaxanthin rapidly and linearly increased until 3 h after incubation, and then gradually increased until 24 h. The concentration of siphonaxanthin in the medium decreased with increasing incubation time (Figure 2A). 

The representative high-performance liquid chromatography (HPLC) chromatogram of an extract of Caco-2 cells after 24 h of incubation with micelles containing siphonaxanthin is shown in Figure 2B. The most predominant peak at approximately 30 min (peak 4) was completely fitted with the peak corresponding to the siphonaxanthin standard. Peak 4 was identified as siphonaxanthin based on its UV-vis spectrum (Figure 2C) and characteristic ions at a charge ratio (*m/z*) of 583.3990 [M + H-H_2_O]^+^, 565.3929 [M + H-2H_2_O]^+^, and 547.3805 [M + H-3H_2_O]^+^ by atmospheric pressure chemical ionization (APCI) (Figure 2D) and 623.4 [M + Na]^+^ by electrospray ionization (ESI). Peak 4’ was identified as *cis*-siphonaxanthin because its APCI-mass spectrometry (MS) spectrum showed the same ion peaks as siphonaxanthin and its UV-vis spectrum presented a *cis* peak at 331 nm (Figure 2C). Except for peaks 4 and 4’, no additional peaks were detected during incubation for 1–6 h (data not shown). However, after incubation for 24 h with siphonaxanthin, peaks 2, 3, and x appeared in the HPLC chromatogram of the cell extract (Figure 2B). The UV-vis spectrum of peak 2 with maximum absorbance at 452 nm was almost consistent with that of siphonaxanthin. Peak 2 showed ion peaks at *m/z* 599.3327, 581.3953, and 563.4973 in the APCI-MS spectrum and *m/z* 621.4 in the ESI-MS spectrum. Peak 3 at 28 min was detected in the cell extract (Figure 2B). The maximum absorbance of peak 3 was not significantly shifted when compared with that of siphonaxanthin (Figure 2C). The APCI-MS spectrum of peak 3 showed two fragment ions at *m/z* 581.3872 and 563.3562; however, the ion peak at *m/z* 599.4 was not observed. The *m/z* values of two compounds (peak 2 and 3) reduced 2 mass compared with these of siphoanxanthin.

### 2.2. Absorption and Tissue Distribution of Siphonaxanthin in Mice

**Figure 3:**
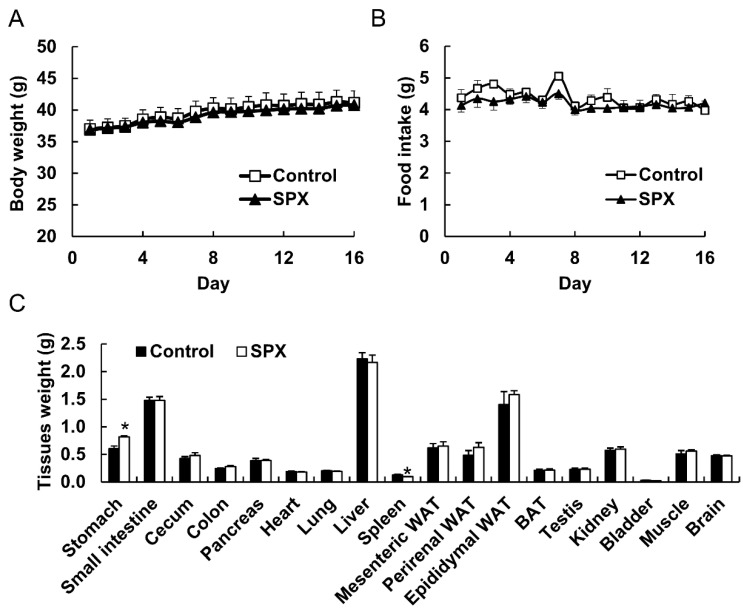
Body weight (**A**), food intake (**B**), and tissue weight (**C**) of ICR mice. ICR mice were fed a control or siphonaxanthin supplementation diet for 16 days. Body weight and food intake were measured daily. After the 16-day feeding period, the mice were killed and their weight was measured. Values are means ± SEM, n = 4. The difference between the control and siphonaxanthin groups was analyzed using the Student’s t-test. * Different from the control group, *p* < 0.05. BAT, brown adipose tissue; WAT, white adipose tissue; SPX, siphonaxanthin.

The body weight of siphonaxanthin fed mice was not altered when compared to the body weight of mice fed the control diet (Figure 3A). During the 16-day experimental period, food intake was not altered between the control and siphonaxanthin groups (Figure 3B). Siphonaxanthin supplementation significantly decreased the weight of the spleen when compared to the control diet (Figure 3C). The weight of the other tissues of siphonaxanthin fed mice did not significantly decrease when compared to that of mice fed the control diet (Figure 3C). 

**Figure 4:**
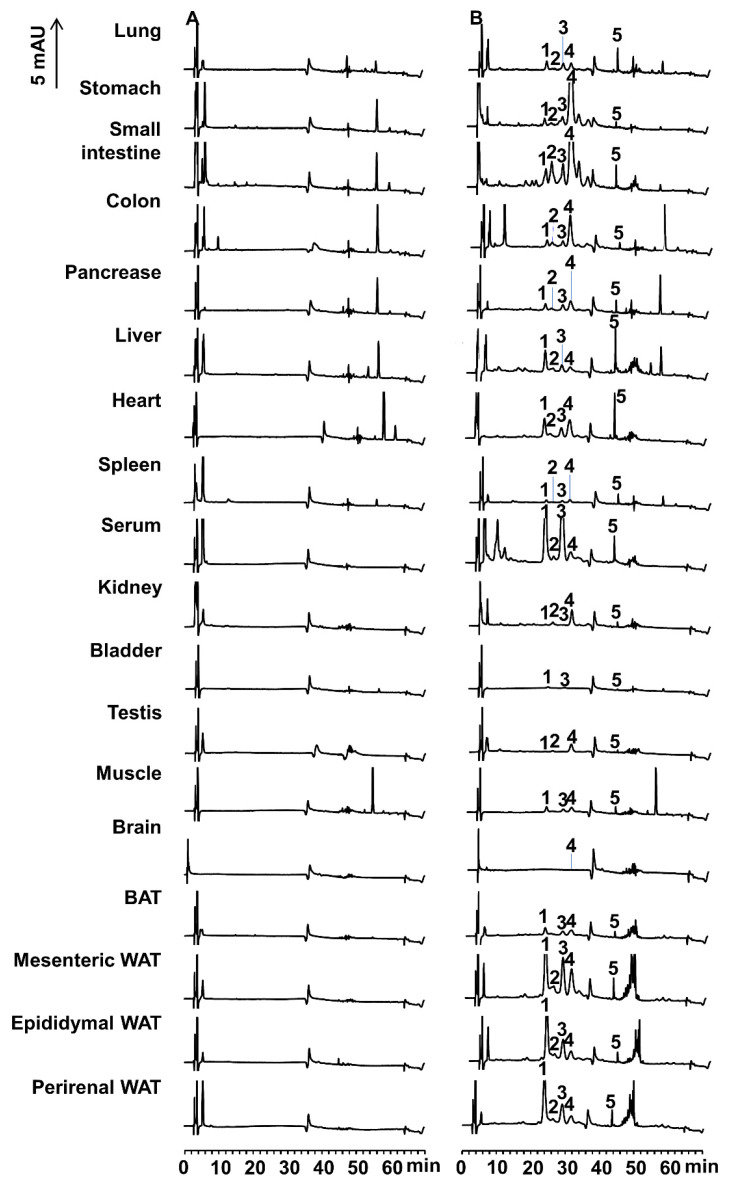
Representative HPLC chromatograms of the extracts from each tissue of mice fed a diet without (**A**) or with (**B**) siphonaxanthin for 16 days. HPLC analysis was performed as described in the experimental methods. The detection wavelength was 450 nm. Peaks with the same number in different chromatograms show similar UV-vis and MS spectra, as shown in Figure 5A,B. 1, 2, 3, and 5, unknown metabolites; 4, siphonaxanthin; Retention time: peak 1 at 22 min, peak 2 at 25 min, peak 3 at 28 min, peak 4 at 30 min and peak 5 at 43 min.

**Figure 5:**
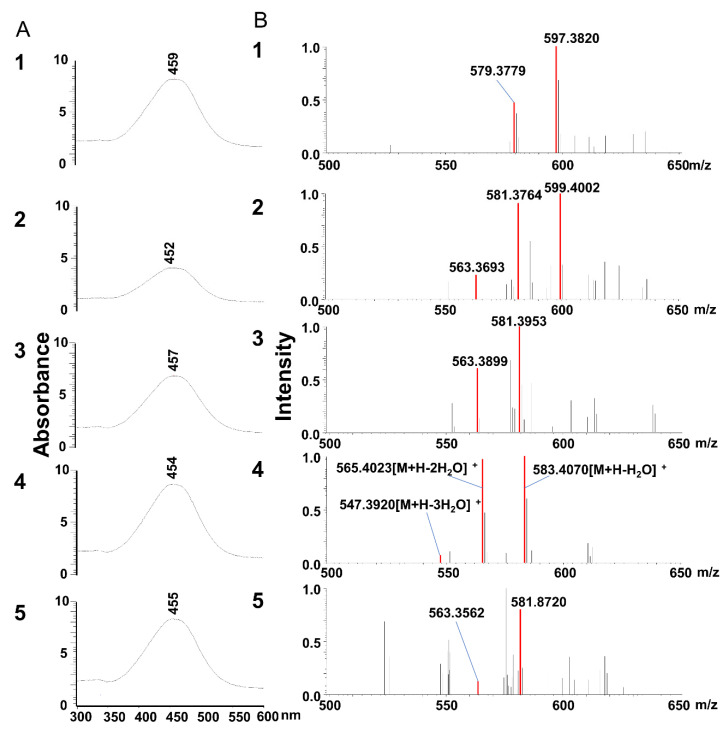
UV-vis spectra (**A**) and APCI-MS spectra (**B**) of peaks 1, 2, 3, 4, and 5 are shown in Figure 4. LC-MS (APCI) analysis was performed as described in the experimental methods.

The representative HPLC chromatograms of the extract from each tissue of mouse fed a diet containing siphonaxanthin for 16 days are shown in Figure 4. Peaks 1, 2, 3, 4, and 5 appeared in the HPLC chromatogram of extracts obtained from most mice tissues; however, these peaks were not detected in control mice. Peak 4 at about 30 min was identified as siphonaxanthin based on its UV-vis and MS spectra (Figure 5). Siphonaxanthin was widely distributed in blood and tissues, except in the bladder. The maximum absorbance of peak 1 at 459 nm shifted 5 nm when compared with that of siphonaxanthin (Figure 5A). Peak 1 showed ion peaks at *m/z* 597.3820 and 579.3779 in the APCI-MS spectrum (Figure 5B) and *m/z* 619.4 in the ESI-MS spectrum. The *m/z* values of peak 1 compound reduced 4 mass compared with these of siphoanxanthin. Peak 2 showed ion peaks at *m/z* 599.4002, 581.3764, and 563.3693 in the APCI-MS spectrum and *m/z* 621.4 in the ESI-MS spectrum. The maximum absorbance of peak 3 was not significantly shifted when compared with that of siphonaxanthin (Figure 5). The APCI-MS spectrum of peak 3 showed two fragment ions at *m/z* 581.3953 and 563.3899; however, the ion peak at *m/z* 599 was not observed. The *m/z* values of peak 2 and 3 reduced 2 mass compared with these of siphoanxanthin. The UV-vis spectrum of peak 5 with maximum absorbance at 455 nm was almost consistent with that of siphonaxanthin (Figure 5A). As the APCI-MS spectrum showed ion peaks at *m/z* 581.8720 and 563.3562 and its retention time was longer than 46 min (Figure 5B). 

### 2.3. Tissue Distribution and Accumulation of Siphonaxanthin and Its Metabolites in Mice

At the end of this feeding study, a higher concentration of intact siphonaxanthin was detected in the small intestine, stomach, and colon than in the plasma and other tissues (Table 1). Notably, the concentration of siphonaxanthin in the mesenteric white adipose tissue was higher than that in the epididymal and perirenal WAT (Table 1). Furthermore, siphonaxanthin was detectable in the mouse brain, although the concentration was extremely low (Table 1).

In addition to siphonaxanthin, peaks 1, 2, and 3 were quantified using the standard curve of siphonaxanthin (Table 1). Following dietary supplementation with siphonaxanthin, the concentration of the compound corresponding to peak 2 was relatively higher in the small intestine than in the mesenteric WAT. The compound corresponding to peak 3 preferentially accumulated in the small intestine followed by the plasma, WATs, and liver. The compound corresponding to peak 1 mainly accumulated in mesenteric WAT followed by the small intestine, plasma, liver, and perirenal and epididymal WATs. However, the compounds of peaks 1, 2 and 3 failed to accumulate in the mouse brain. The proportion of peak 1 in the liver and WATs was 40–60% of total carotenoids, while the proportion was less than 10% in the small intestine, stomach, and colon, and less than 5% in the kidney and testis (Table 1). In all tissues, the proportion peak 2 was limited and less than 15% of the total carotenoids (Table 1). The proportion of peak 3 in the liver and WATs was more than 20–27% of total carotenoids, while the proportion was less than 10% in the small intestine, stomach and colon, and less than 2% in the kidney and testis (Table 1). 

## 3. Discussion

To enhance understanding of the potential health benefits of dietary carotenoids, studies regarding their bioavailability and biotransformation are crucial. In this study, the absorption, distribution, and accumulation of dietary siphonaxanthin were investigated in differentiated Caco-2 cells and ICR mice.

At incubation with siphonaxanthin for 24 h in the Caco-2 cells, some unknown peaks in HPLC chromatography were appeared. The *m/z* values of two compounds (peaks 2 and 3) reduced 2 mass compared with these of siphoanxanthin. Thus, we speculated that siphonaxanthin might be oxidized into two types of didehydro-metabolites in differentiated Caco-2 cells. It has been suggested that siphonaxanthin is derived from lutein via an oxidative pathway in green algae [10]. Reportedly, metabolites of lutein, keto-carotenoids, were detected in the plasma and tissues of mice and humans [22]. Owing to the similar chemical structures of lutein and siphonaxanthin, it was assumed that the possible structures of the two siphonaxanthin metabolites found in Caco-2 cells were the dehydrogenization products of siphonaxanthin, in which a hydroxyl group could be oxidized into a keto group. Siphonaxanthin has three hydroxyl groups at positions 3, 19, and 3’, all of which may be oxidized into the keto group. Previously, the oxidation of lutein at the 3 and 3’ position [22], the oxidation of capsanthin at the 3’ position to capsanthone [23], and the dehydrogenation of fucoxanthinol at position 3 to amarouciaxanthin A in mice liver [24] has been reported after ingestion of these carotenoids in vivo. These reports indicate that a hydroxyl group at the end group would be liable to be oxidized. Thus, the two dehydrogenated metabolites of siphonaxanthin were presumed to be the 3’-didehydro-metabolite and 3-didehydro-metabolite of siphonaxanthin. In the future, we plan to identify the differences between these two metabolites. 

Two compounds of peaks 2 and 3 were present in both Caco-2 cells and the small intestine, siphonaxanthin might be converted to the above didehydro-metabolites of siphonaxanthin by some enzymes associated with oxidation reactions in the small intestine of mice. These two compounds were observed in the plasma and other tissues. Additionally, in most tissues, the compound of peak 3 (Figure 4) was more abundant than peak 2 (Figure 4), especially in the plasma and WATs (Table 1), indicating that the structure of the compound of peak 3 might be more stable in vivo. Lutein has 3-hydroxy β-end and 3’-hydroxy ε-end groups. The former is preferentially oxidized into 3-didehydro ε-end [25]. It has been reported that the 3-didehydro-metabolite of lutein (3’-hydroxyl-ε,ε-caroten-3-one), found to be largely accumulated in mouse tissues, is more stable than the 3′-didehydro-metabolite of lutein (3-hydroxyl-β,ε-caroten-3’-one), detected in the human serum [22]. Furthermore, the 3-hydroxy β-end group in lutein is converted to a 3-didehydro ε-end group via the 3-didehydro β-end group, when incubated in a mixture of lutein with a post-mitochondrial fraction of mouse liver [25]. The intermediates containing the 3-didehydro β-end group, produced by dehydrogenation, demonstrate unstable conformations, thus resulting in structural isomerization into an ε-end group by double-bond migration [25]. However, the intermediates containing the 3-didehydro β-end group are not detected after the intake of lutein [25]. Therefore, we presumed that the structure of the putative final 3-didehydro-metabolites of siphonaxanthin might include the 3-dehydroxy ε-end group but not the β-end group because the β-end group is unstable. Based on the above discussion, peak 2, which demonstrated minimal accumulation in most tissues except for testis and kidney, might also be an unstable 3-didehydro-metabolite of siphonaxanthin, containing a 3-didehydroxy β-end group or a 3′-didehydro-metabolite of siphonaxanthin. In contrast, peak 3, with abundant accumulation, was presumed to be a 3-didehydro-metabolite of siphonaxanthin. 

The most abundant metabolite eluted at 22 min (peak 1) accounted for 40–60% of siphonaxanthin and its metabolites in plasma, liver and WATs. Based on the MS spectra, this metabolite was also considered as a dehydrogenization product of siphonaxanthin. It is speculated that two hydroxyl groups were transformed into two keto groups in the structure of the compound. Reportedly, lutein is oxidized into ε,ε-carotene-3,3’-dione in mice [22], and 4,4’-dimethoxy-β-carotene is oxidized into 4,4’-diketo-β-carotene in human subjects [26]. Thus, the metabolite eluted at 22 min might be identified as a 3,3’-tetradehydro-metabolite of siphonaxanthin. And it was not possible to directly analyze the small amounts of crude metabolites of siponaxanthin found in each tissue by NMR. In the future, we will isolate these compounds and reveal the structure of the oxidized products of siphonaxanthin, especially the location of the carbonyl groups.

Based on the comparison with the other carotenoids which have similar structures with siphonaxanthin, the change of mass, and also not significantly shifted the maximum absorbance of UV-vis spectra, we speculated the compounds of peaks 1, 2 and 3 to be the dehydro-metabolites of siphonaxanthin. And thus, we speculated the formulas of peaks 1, 2 and 3 (Supplemental Table S1). Based on these predicted formulas, the predicted ion and the observed ion are shown in the Supplemental Table S1. However, the error between the predicted ion and the observed ion was always large (2–39 ppm). Peak 4 were identified as siphonaxanthin compared with the standard, however, the error of peak 4 was also large. Thus, we considered that the error was caused by the factors of environment, temperature, and the instrument itself. However, another possibility is that real formulas of peaks 1, 2 and 3 might not be our predicted formulas. In the future, we will verify this using the internal calibration method.

Furthermore, the carotenoids eluted at approximately 43–52 min in cells and mice were assumed to be the esters of siphonaxanthin. In differentiated Caco-2 cells, peridinin is converted to peridiniol and its fatty acid esters [27]. No information is available regarding the fatty acid esters of carotenoids in mice. At present, due to the coexistence of large amounts of triacylglycerols in the extract from cells and mice, the identification of the metabolites is challenging and needs to be addressed in future studies. The metabolic pathways of lutein and zeaxanthin have been suggested in many reports [26,28,29]. 

The metabolites of fucoxanthin, such as fucoxanthinol and amarouciaxanthin A, mainly accumulate in adipose tissues [30]. Additionally, it has been reported that lutein [22] and β-carotene [31,32] mainly accumulate in the liver instead of adipose tissues. In the present study, siphonaxanthin was not completely converted to metabolites in the gastrointestinal tract, and an unchanged form of siphonaxanthin was also detected in the plasma and most tissues, except the bladder, after the 16-day feeding period, along with its metabolites. Canthaxanthin, lycopene, and β-carotene are known to accumulate to a small extent in the colon, small intestine, stomach, and large intestine [33,34,35]. Siphonaxanthin might merely stick to the intestinal mucosa, rather than accumulate in the intestine. Notably, siphonaxanthin accumulated more easily in the mesenteric adipose tissue than epididymal WAT, perirenal WAT, and BAT, which might be attributed to the fact that the mesenteric WAT is closer to the gastrointestinal tract. These results are consistent with our previous findings that siphonaxanthin mainly accumulates in the mesenteric WAT and significantly inhibits the lipid accumulation of mesenteric WAT in KK-Ay mice administrated siphonaxanthin for 6 weeks. In our previous study, siphonaxanthin and its metabolites were not separated, and thus the total accumulation of siphonaxanthin and its metabolites in the mesenteric WAT was 649 pmol/g [16]. In this study, although the experimental conditions differed, the total accumulation of siphonaxanthin and its presumed metabolites reached approximately 70% of the above stated 649 pmol/g. However, the accumulation level did not change the weight of mesenteric WAT in ICR mice. Thus, we speculated that siphonaxanthin might have low toxicity and fewer side effects, such as excessive weight loss, in normal mice. To evaluate the effect of siphonaxanthin and its metabolites on normal subjects, a longer feeding period should be undertaken in the future.

The distribution of each possible metabolite depended on the tissues. The compounds corresponding to peak 3 and peak 1 were more abundant than siphonaxanthin in the liver, serum, and WATs, whereas the proportion of these two compounds was inversed in other evaluated tissues. The compound corresponding to peak 2 mainly accumulated in the small intestine, with small amounts observed in the other tissues. The tissue-dependent distribution of presumed siphonaxanthin metabolites might be associated with the tissue-specific distribution of enzymes or the rate of metabolism and transport of each metabolite. 

In general, the bioavailability of functional food ingredients in diets can affect their biological effectiveness. The bioavailability of dietary carotenoids depends on several steps, including absorption in the intestinal epithelia and metabolism. In this study, siphonaxanthin was absorbed and metabolized by mice. In contrast with mice, humans tend to accumulate carotenoids selectively, possibly through discriminative uptake and/or re-excretion by intestinal cells and metabolism in the body [28]. When siphonaxanthin, solubilized in the mixed micelles compatible with those formed in the intestine, were incubated with human intestinal Caco-2 cells, the accumulation of siphonaxanthin increased. The results demonstrated that siphonaxanthin might be absorbed and metabolized by humans. Our previous study shows that the content of siphonaxanthin in *C. cylindricum* harvested in autumn is approximately 230 μg/g dry weight [18]. In this study, 65.79 nmol siphonaxanthin/g of diet (0.004%) is equivalent to a daily dosage of approximately 18 g dried *C. cylindricum* powder/kg body weight. Thus, purified siphonaxanthin is useful as a dietary supplement. In the future, it is necessary to investigate the behavior of siphonaxanthin in human subjects. 

In addition to intestinal absorption, tissue distribution and metabolism also affect the bioavailability of carotenoids. The present study indicates that dietary siphonaxanthin might be partly dehydrogenated after absorption, and both intact and metabolized forms of siphonaxanthin could accumulate in the body. To understand the bioavailability of siphonaxanthin, further efforts are needed to clarify the metabolic pathway, metabolic rate, and the enzymes involved in the oxidative transformation of siphonaxanthin. In some tissues and plasma, the accumulation of the oxidative metabolites was much greater than that of intact siphonaxanthin; therefore, to elucidate the role of siphonaxanthin in human health, the bioactivity of siphonaxanthin metabolites is worth evaluating. 

In summary, siphonaxanthin was absorbed accumulated in the mice. Three possible metabolites of siphonaxanthin were also found in ICR mice. The distribution of siphonaxanthin and its presumed metabolites depended on the tissue. Although further studies are needed to elucidate the metabolic mechanisms of siphonaxanthin and identify the structure of metabolites of siphonaxanthin, this study has provided useful information for developing siphonaxanthin applications beneficial to human health. In particular, this work provides important reference information to understand the bioavailability and tissue accumulation of other carotenoids with similar structures.

## 4. Materials and Methods

### 4.1. Preparation of Siphonaxanthin

Siphonaxanthin was extracted from green alga (*Codium cylindricum*) as previously described [16]. The extracted carotenoid was purified by HPLC (LC-6, Shimadzu, Kyoto, Japan) and the purified siphonaxanthin (purity > 98%) was used. Siphonaxanthin was stored at −80 °C until further use. 

### 4.2. Cell Culture

Caco-2 cells obtained from the RIKEN Gene Bank (Tsukuba, Japan) were cultured in Dulbecco’s Modified Eagle’s Medium (DMEM) containing 10% fetal bovine serum (FBS), 1% penicillin-streptomycin (PS), and 1% non-essential amino acids in a humidified atmosphere of 95% air and 5% CO_2_ at 37 °C. For differentiation, cells were seeded in 12-well plates at a density of 2.0 × 10^5^ cells/well in 1 mL DMEM medium described above and allowed to differentiate until day 22 from seeding. The medium was regularly changed three times a week. Experiments were performed at day 22 post-seeding on 12-well plates. 

### 4.3. Treatment of Caco-2 Cells with Micellar Siphonaxanthin

Siphonaxanthin solubilized in micelles was added to the medium for treatment, and micelle formation was performed as previously described [36]. Briefly, sodium taurocholate, monoolein, oleic acid, lysophosphatidylcholine, and siphonaxanthin dissolved in dichloromethane or methanol were mixed using a vortex mixer, and the organic solvent was evaporated using nitrogen gas. The residue was then dissolved in serum-free DMEM. The final concentration of each component in the medium was adjusted to 2 mmol/L sodium taurocholate, 100 μmol/L monoolein, 33.3 μmol/L oleic acid, 50 μmol/L lysophosphatidylcholine, and 1.0 μmol/L siphonaxanthin. The resultant solution should be optically clear. This medium was filtered using a 0.22 μm filter before supplementation to the culture cell. The concentration of micellar carotenoid was determined as 1.0 ± 0.05 μmol/L by HPLC before use in the following experiment.

The cell monolayers on 12-well plates were rinsed with the serum-free medium and then incubated in 1 mL of medium containing micellar siphonaxanthin. After incubation for 0, 1, 3, 6, and 24 h, the medium was collected, and the cells were washed twice with ice-cold phosphate-buffered saline (PBS) containing 10 mmol/L sodium taurocholate to remove surface-bonded carotenoids, followed by an additional washing with PBS. The cells were scraped into PBS and then centrifuged at 1000× *g* at 4 °C for 5 min. The cell pellets were resuspended in 0.5 mL PBS and homogenized with a sonicator (Qsonica Q55). To extract siphonaxanthin, 0.4 mL of the cell homogenate was mixed with 1.5 mL of dichloromethane/methanol (1:2, v/v), and vigorously vortexed. Then, 0.75 mL of hexane was added to the mixture, followed by strong agitation, and centrifugation at 1690× *g* at 4 °C for 10 min. The upper organic phase was transferred to a fresh test tube, the sample was extracted again by adding 0.5 mL of dichloromethane, and then 0.75 mL of hexane. This extraction was repeated three times. All organic phases were pooled together and evaporated gently under a nitrogen stream. The residue was dissolved in methanol and subjected to quantitative and qualitative analysis. Because there were no other carotenoids except for siphonaxanthin were observed at 6, 12, and 18 h, the quantitative analysis of the cell extraction at all time points was performed using a photodiode array detector (SPD-M20A, Shimadzu, Kyoto, Japan) connected to the HPLC system (LC-6, Shimadzu, Kyoto, Japan). The qualitative analysis of the cell extraction at 24 h was performed using LC-MS system as described below. The concentration of siphonaxanthin in the medium was also analyzed. An aliquot of the medium was mixed with 4-fold methanol and subjected to the above HPLC analysis for quantify.

### 4.4. Animal Studies

All experimental animal protocols were approved by the Animal Experimentation Committee of Kyoto University, Japan, for the care and use of experimental animals (Approval No. 26–35). Male ICR mice (7 weeks of age) were purchased from Japan SLC, Inc. (Hamamatsu, Japan). All mice were housed individually and maintained on an alternating 12-h light/dark cycle at a temperature of 23 ± 1 °C and free access to drinking water and chow (Oriental Yeast Co., Ltd., Tokyo, Japan). After an acclimatization period of 1 week, the mice were randomly divided into control and siphonaxanthin groups (n = 4 per group). Mice in the control group were fed an AIN-93G diet. The siphonaxanthin mouse group was fed the AIN-93G diet with siphonaxanthin supplementation, 65.79 nmol/g of diet (0.004%). The total food intake and body weight were recorded daily. After dietary supplementation for 16 days, the mice were anesthetized with isoflurane. Blood was collected from the caudal vena cava. Plasma was prepared by centrifuging at 400 *g* for 15 min at 4 °C. The tissues were rapidly removed, weighed, and immediately frozen in liquid nitrogen, and stored at −80 °C until use. 

Carotenoids were then extracted from the plasma and tissues. Briefly, aliquots of tissue samples (0.2 g) were homogenized in a 9-fold volume of 0.9% NaCl saline with a homogenizer dispenser (T10 basic ULTRA-TURRAX, IKA). Plasma samples were diluted with a 3-fold volume of Milli-Q water. The resultant tissue homogenates (0.9 mL) or diluent plasma samples (0.9 mL) were mixed with 3 mL of dichloromethane/methanol (2:1, v/v) to extract carotenoids. The samples were extracted three times, and the dichloromethane layer was collected after centrifugation at 1690 *g* at 4 °C for 10 min. After evaporation under nitrogen, the residue was resuspended in 20 μL of dichloromethane and methanol (1:1, v/v) for HPLC analysis. 

### 4.5. HPLC and MS Analysis

HPLC analysis was performed using a Prominence LC system (Shimadzu, Kyoto, Japan) connected to a photodiode array detector (SPD-M20A, Shimadzu, Kyoto, Japan), followed by an ion trap-time of flight mass spectrometer (LCMS-IT-TOF, Shimadzu, Kyoto, Japan) equipped with an atmospheric pressure chemical ionization (APCI) source or electrospray ionization (ESI) source. Siphonaxanthin was separated on a TSK gel ODS-80Ts QA column (2.0 × 250 mm, 5 μm, Tosoh, Tokyo, Japan). The binary gradient mobile phase was methanol/water (83:17, v/v) containing 0.1% ammonium acetate as mobile phase A and ethyl acetate/methanol (30:70, v/v) containing 0.1% ammonium acetate as mobile phase B. The column was eluted at a flow rate of 0.2 mL/min using the following gradients: 0–30 min, 0% B; 30–45 min, 0–100% B; 45–60 min, 100% B; 60–65 min, 100–0% B; 65–70 min, 0% B. Siphonaxanthin was detected at 450 nm. The APCI source was heated at 200 °C, and the probe was maintained at 400 °C. Nitrogen was used as sheath gas at 2.0 L/min, and drying gas was used at 25 kPa. Mass spectra were recorded in the positive ion mode. For the ESI source, sheath gas was set at 1.5 L/min, and drying gas was used at 120 kPa. A spray voltage of 4.5 kV was used for positive ionization. The peak identities of siphonaxanthin and its possible metabolites were further confirmed from their characteristic UV-vis spectra and their positive ions. Siphonaxanthin was quantified from its peak area at 450 nm using an external standard calibration curve with purified siphonaxanthin. Due to the unavailability of standards, possible metabolites of siphonaxanthin were estimated from the siphonaxanthin standard curve [22]. 

### 4.6. Statistical Analysis

Data analyses were performed using the statistical program SPSS 16.0 for Windows (SPSS Inc., Chicago, IL, USA). Changes in the concentration of siphonaxanthin in Caco-2 cells were analyzed by 1-factor ANOVA with repeated measures. Data are represented as mean ± SEM. Statistical significance was defined as *p* < 0.05.

## Figures and Tables

**Table 1:** Concentration of siphonaxanthin and compounds corresponding to peak 1, 2, and 3 in the plasma and tissues of mice at the end of the 16-day dietary supplementation with siphonaxanthin ^1^.

Tissue	Peak 1	Peak 2	Peak 3	Siphonaxanthin	Total
Stomach, pmol/g (%)	26.6 ± 13.5 (1.2)	19.0 ± 3.3 (0.9)	86.1 ± 11.0 (3.9)	2054 ± 636 (94.0)	2186 ± 612
Small intestine, pmol/g (%)	172 ± 114 (4.0)	287 ± 132 (6.7)	423 ± 273 (9.8)	3428 ± 1389 (79.5)	4310 ± 1837
Pancreas, pmol/g (%)	24.7 ± 14.3 (24.3)	6.4 ± 3.8 (6.3)	24.4 ± 14.1 (24.0)	46.1 ± 12.5 (45.4)	102 ± 44.2
Spleen, pmol/g (%)	10.5 ± 5.2 (23.2)	2.9 ± 1.0 (6.4)	11.1 ± 4.9 (24.7)	20.6 ± 3.4 (45.7)	45.1 ± 12.5
Colon, pmol/g (%)	12.3 ± 7.5 (5.1)	8.7 ± 0.4 (3.6)	20.9 ± 11.4 (8.7)	198 ± 24.5 (82.5)	239 ± 11.3
Lung, pmol/g (%)	18.3 ± 10.8 (30.9)	0.6 ± 0.4 (1.0)	17.3 ± 10.0 (29.3)	22.9 ± 8.2 (38.8)	59.0 ± 29.3
Heart, pmol/g (%)	35.0 ± 20.2 (27.2)	4.6 ± 2.6 (3.5)	21.9 ± 12.7 (17.0)	67.1 ± 25.2 (52.2)	129 ± 60.6
Liver, pmol/g (%)	110 ± 63.4 (55.3)	11.3 ± 4.6 (5.7)	40.3 ± 21.2 (20.3)	37.1 ± 14.4 (18.7)	198 ± 102
Bladder, pmol/g (%)	14.6 ± 8.5 (58.5)	N.D.	10.4 ± 6.1 (41.5)	N.D.	25.0 ± 14.6
Muscle, pmol/g (%)	9.9 ± 5.7 (36.5)	0.7 ± 0.7 (2.6)	2.2 ± 2.2 (8.3)	14.2 ± 7.5 (52.6)	27.1 ± 15.1
Plasma, pmol/mL (%)	162 ± 93.4 (48.8)	3.5 ± 2.0 (1.0)	147.4 ± 78.6 (44.6)	18.3 ± 4.0 (5.5)	331 ± 177
BAT, pmol/g (%)	25.3 ± 15.4 (34.6)	0.6 ± 0.6 (0.8)	16.7 ± 9.5 (22.9)	30.5 ± 11.4 (41.7)	73.1 ± 36.0
Mesenteric WAT, pmol/g (%)	188 ± 106 (41.8)	43.6 ± 21.8 (9.7)	108.1 ± 58.7 (24.0)	110 ± 50.8 (24.5)	449 ± 236
Perirenal WAT, pmol/g (%)	95.5 ± 62.2 (57.4)	4.3 ± 2.5 (2.6)	44.4 ± 29.4 (26.7)	22.1 ± 12.0 (13.3)	166 ± 105
Epididymal WAT, pmol/g (%)	112 ± 69.0 (58.9)	N.D.	50.6 ± 30.3 (26.5)	27.8 ± 14.3 (14.6)	190 ± 113
Kidney, pmol/g (%)	0.5 ± 0.5 (1.0)	6.6 ± 2.9 (14.6)	0.1 ± 0.1 (0.3)	38.2 ± 15.7 (84.1)	45.4 ± 18.9
Testis, pmol/g (%)	1.2 ± 0.8 (4.1)	3.0 ± 1.8 (10.2)	0.4 ± 0.4 (1.3)	25.0 ± 10.0 (84.3)	29.6 ± 12.6
Brain, pmol/g (%)	N.D.	N.D.	N.D.	0.4 ± 0.3 (100)	0.4 ± 0.3

^1^ The quantification of siphonaxanthin and metabolites was based on the peaks (shown in Figure 4) in the HPLC chromatogram. Siphonaxanthin and metabolites were quantified using the standard curve of siphonaxanthin. The values in parenthesis for siphonaxanthin (peak 4) and metabolites (peak 1–3) are also expressed as % of the total carotenoids. Values are means ± SEM, n = 4. BAT, interscapular brown adipose tissue; WAT, white adipose tissue; N.D., not detected.

## Supplementary Materials

The following are available online at https://www.mdpi.com/1660-3397/18/6/291/s1, Table S1: Maximum absorbance of UV-vis spectra and LC–MS data in the positive ion mode of compounds identified in small intestine samples of mice at the end of the 16-day dietary supplementation with siphonaxanthin.
