# Supplementary material for: Absorption and Tissue Distribution of Siphonaxanthin from Green Algae
Source: Mar Drugs. 2020 Jun 1;18(6):291. doi: 10.3390/md18060291 (PMC7345836; doi:10.3390/md18060291)
Supplement: Supplementary file 1 [file marinedrugs-18-00291-s001.pdf]

marine drugs

MDPI

Supplemental Table 1. Maximum absorbance of UV-vis spectra and LC–MS data in the positive ion mode of compounds identified in small intestine samples of mice at the end of the 16-day dietary supplementation with siphonaxanthin.

Peak number	Compound	Maximum absorbance (nm)	Formula[1]	Rentention time (min)	Predicted Ion	Predicted m/z	Observed m/z	Error (ppm)
1	Unidentified	459	$C_{40}H_{52}O_4$ predicted	22	$[M+H]^+$	597.3938	597.3820	-19.75
					$[M+H-H_2O]^+$	579.3833	579.3779	-9.32
2	Unidentified	452	$C_{40}H_{54}O_4$ predicted	25	$[M+H]^+$	599.4095	599.4002	-15.52
					$[M+H-H_2O]^+$	581.3989	581.3764	-38.70
					$[M+H-2H_2O]^+$	563.3884	563.3693	-33.90
3	Unidentified	457	$C_{40}H_{54}O_4$ predicted	28	$[M+H-H_2O]^+$	581.3989	581.3953	-6.19
					$[M+H-2H_2O]^+$	563.3884	563.3899	2.66
4	Siphonaxanthin	454	$C_{40}H_{56}O_4$	30	$[M+H-H_2O]^+$	583.4146	583.4070	-13.03
					$[M+H-2H_2O]^+$	565.4040	565.4023	-3.01
					$[M+H-3H_2O]^+$	547.3934	547.3920	-2.56

[1] The formulas of peaks 1, 2 and 3 were speculated based on the previous reports of the other carotenoids (such as lutein and zeaxanthin) which have similar structures with siphonaxanthin.
